# A Student Pharmacist Quality Engagement Team to Support Initial Implementation of Comprehensive Medication Management within Independent Community Pharmacies

**DOI:** 10.3390/pharmacy8030141

**Published:** 2020-08-09

**Authors:** Sophia M. C. Herbert, Joni C. Carroll, Kim C. Coley, Stephanie Harriman McGrath, Melissa Somma McGivney

**Affiliations:** 1 School of Pharmacy, University of Pittsburgh, Pittsburgh, PA 15213, USA; sophia.cothrel@pitt.edu (S.M.C.H.); joni.carroll@pitt.edu (J.C.C.); coley@pitt.edu (K.C.C.); 2 Pennsylvania Pharmacists Care Network, Pittsburgh, PA 15213, USA; stephanie.mcgrath@pitt.edu

**Keywords:** student pharmacists, CPESN, quality improvement, implementation science, medication therapy management, comprehensive medication management, community pharmacy services

## Abstract

In 2017, the Pennsylvania Pharmacists Care Network (PPCN), an enhanced pharmacy services network, and STRAND Clinical Technologies partnered to launch a payor contract with a Pennsylvania Medicaid Managed Care Organization for the provision of comprehensive medication management (CMM) at PPCN pharmacies. PPCN and the Community Leadership and Innovation in Practice Center at the University of Pittsburgh School of Pharmacy partnered to create the Quality Engagement Team (QET), a group of student pharmacists formed with the intent to support the initial implementation of this contract. The QET supported the pharmacies through biweekly phone calls, which led to increased pharmacist engagement and produced impactful patient encounter stories that were then reported back to the payor. We utilized Active Implementation Frameworks and select implementation strategies from the Expert Recommendations for Implementing Change project throughout the implementation period. The QET supported the successful implementation of this payor contract, which saw an increase in CMM encounters completed by the pharmacists during each month of the four-month contract period. Students, pharmacists and the payor each derived meaningful benefits from this initiative. Student pharmacists can be a powerful asset in the implementation of payor programs within an enhanced pharmacy services network, resulting in the mutually beneficial and sustainable support of the network.

## 1. Introduction

### 1.1. Background

The Pennsylvania Pharmacists Care Network (PPCN) is an enhanced pharmacy services network of over 140 community pharmacies in Pennsylvania. PPCN is a local member of the national community pharmacy enhanced services network, CPESN USA. CPESN USA is a clinically integrated network of over 2500 community pharmacies across the United States formed to advance pharmacist-provided care [1,2,3,4]. Clinically integrated networks (CINs) are “a collection of health care providers that demonstrates value to the market by working together to facilitate the coordination of patient care across conditions, providers, settings, and time to improve patient care and decrease overall healthcare costs [1].” There are seven components to CINs: (1) structure and governance; (2) provider leadership; (3) participation criteria; (4) performance objectives and quality improvement; (5) information technology; (6) contracting; (7) distribution of funds [5,6]. PPCN is one of 49 local CPESN networks nationwide with a local governance structure and leadership developed to meet the criteria of CINs.

PPCN’s mission is to enable and support the delivery of quality pharmacy care and outcomes in collaboration with patients, practitioners and stakeholders involved in a patient’s care [7]. PPCN recruits pharmacies that provide enhanced patient care services for their patients beyond dispensing medications [1,8]. In 2017, PPCN partnered with the pharmacy technology solutions company STRAND Clinical Technologies (STRAND) to utilize Strand-Rx, their community pharmacist-driven clinical platform and documentation system that utilizes the Pharmacist eCare Plan (PeCP) [9]. The PeCP is “an interoperable standard that allows for pharmacy technology providers to have a common method of exchanging information related to care delivery, including patient goals, health concerns, active medication list, drug therapy problems, laboratory results, vitals, payer information and billing for services [10].” Strand-Rx was one of the first clinical platforms for pharmacists that facilitated the documentation and submission of eCare plans [10,11].

In September 2017, PPCN and STRAND launched their first payor contract with a Pennsylvania Medicaid Managed Care Organization (MCO), which covers three of five Pennsylvania Medicaid regions. There were 61 independent pharmacies that self-selected to participate in this first payor contract. This initial four-month contract period compensated community pharmacists for providing comprehensive medication management (CMM) to Medicaid members who filled at least one medication at a PPCN pharmacy. CMM is defined as “the standard of care that ensures each patient’s medications [...] are individually assessed to determine that each medication is appropriate for the patient, effective for their medical condition, safe given the comorbidities and other medications being taken, and able to be taken by the patient as intended. CMM includes an individualized care plan that achieves the intended goals of therapy with appropriate follow-up to determine actual patient outcomes” [12,13]. Through the PeCP standard, the Strand-Rx platform served as a place to document patient care provided and facilitated billing for these encounters. The contract permitted pharmacists to provide and be compensated for CMM that encompassed one initial patient encounter and up to six follow-up patient encounters within a calendar year for an individual patient.

There is limited evidence and guidance on how to implement a payor contract for CMM in community pharmacies within a CIN, and in 2017, guidance was even more sparse [3,4]. Quality assurance and improvement are critical components of implementation within a CIN. PPCN and STRAND partnered with the University of Pittsburgh School of Pharmacy students, faculty and staff to pilot a student-led model to support the initial implementation of the payor contract to meet the quality assurance and improvement needs.

### 1.2. Objective

The objective of this paper is to describe how a student-led Quality Engagement Team (QET) within a school of pharmacy supported a local CPESN network during initial implementation of their first payor contract for CMM.

## 2. Innovation

### 2.1. Formation and Structure of the QET

Faculty and staff at the Community Leadership and Innovation in Practice (CLIP) Center at the University of Pittsburgh School of Pharmacy, in partnership with community-based practices and partners, assist with community-based pharmacist innovations, pioneer novel approaches to education, develop collaborative research, and implement sustainable pharmacist-provided care models [14]. Student pharmacists are integral to these efforts and may elect to participate through several mechanisms, including the CLIP Center Area of Concentration (ARCO). The CLIP ARCO provides PharmD students an opportunity to participate in (1) elective coursework on advancing outpatient pharmacist practices; (2) a community-based research course; and (3) a longitudinal research or quality improvement project. These projects immerse students in practice transformation efforts that provide hands-on learning opportunities to advance community pharmacy practice [14].

The University of Pittsburgh School of Pharmacy and PPCN have partnered since PPCN’s inception. In 2017, the School and PPCN collaborated to create the QET. The QET was comprised of five University of Pittsburgh School of Pharmacy student pharmacists who supported the initial implementation of the payor contract for CMM. Four of these students were enrolled in the CLIP ARCO and participated through a longitudinal quality improvement project. Independent pharmacies participating in the payor contract for CMM were contacted by the student-led QET during the four-month contract period for the purposes of quality improvement and to assist with the initial implementation of the contract for CMM.

Both PPCN leadership and CLIP Center faculty and staff supervised the QET and met with the students biweekly throughout the four-month contract period. Supervision activities included, but were not limited to, assisting QET students with ongoing problem solving and serving as mentors for the project. Mentors also directly assisted students with triaging pharmacists’ questions and issues that the students were unable to resolve on their own.

### 2.2. QET Activities

This QET project was designated as quality improvement by the University’s institutional review board. The QET students first contacted pharmacists from each of the participating independent pharmacies to invite them to engage in a series of telephone-based, key informant, semi-structured interviews. Pharmacists who agreed were then contacted biweekly via telephone over a four-month period (September–December 2017) by an individual QET student. Students were assigned to call the same pharmacies during the initial implementation so that they could develop a rapport with participating pharmacists. If pharmacists were unable to be reached by telephone, they were called again at a later time. The purpose of these calls during the initial implementation phase was to elicit information on (1) the pharmacy teams’ experience with integrating CMM into workflow; (2) involving members of the pharmacy team in CMM; (3) patient engagement strategies and perceptions of the patient experience with CMM; and (4) successful local implementation strategies and desired implementation support. A semi-structured interview guide (see Table 1) was developed using a combination of investigator-generated questions and expert opinion from PPCN and CLIP Center leadership. The students documented information from their phone calls through a combination of audio-recording and transcribing the phone conversations, and writing field notes during the phone calls.

QET students kept a record of issues that required collective troubleshooting and a list of questions from pharmacists that required further assistance from mentors to answer. QET students, PPCN and CLIP Center mentors worked through these issues at biweekly team meetings. This process provided consistent and timely feedback to PPCN leadership from the pharmacists’ phone calls. Students also sent biweekly emails to a primary contact at STRAND which outlined both pharmacist questions and their feedback regarding the Strand-Rx platform and its use for documenting CMM. This communication between the QET students, PPCN leadership and STRAND was critical to rapidly solve problems for the pharmacists providing CMM. For example, within the first few weeks of the call period, one pharmacist discovered that some of the common disease states she saw in her practice were not included in the available list of options for health conditions within Strand-Rx. The pharmacist reported her concerns to a QET student during one of their biweekly calls, and the student fed this concern back to STRAND. By the time the student reconnected with the pharmacist, Strand-Rx was updated to include the health conditions previously missing from the platform. Another example of rapid problem-solving occurred when QET students identified that pharmacists were requesting additional education and support on how to properly engage pediatric patients in CMM. The QET provided this information to the PPCN leadership, which led to the accelerated formation of a pediatric patient guide created by CLIP Center faculty and staff. The open lines of communication between the participating pharmacists, students, STRAND and PPCN team allowed for “close-to-real-time” solutions to daily barriers in implementation.

### 2.3. Utility of the QET

QET students helped pharmacies progress along the implementation continuum. The phone conversations and pharmacists’ questions at the beginning of the four months often centered around operations and technology, whereas towards the end of the four months, pharmacists had questions about documentation, billing and clinical patient care needs. Pharmacist engagement and knowledge of the program increased over time, leading to more in-depth questions, problem solving and idea sharing with QET students. These students captured a wide range of information from the pharmacists during the phone calls. Valuable takeaways from early adopters were disseminated to PPCN membership during regularly scheduled conference calls and via email newsletters so that other participating pharmacy teams could apply and adapt best practices locally.

Once CMM encounters were underway at participating pharmacies, QET students collected patient care stories from pharmacist–patient CMM encounters during their calls. These patient stories demonstrated the high-quality care provided by the pharmacists. For example, one patient had not been filling her medications for several chronic conditions due to misunderstanding her insurance coverage. The pharmacist intervened, educated the patient regarding her insurance coverage, and filled the patient’s chronic medications. If it had not been for this intervention, the patient could have gone months without critical medications. This and other stories helped communicate to the payor the high-quality care provided to their members. Additionally, CLIP Center faculty and staff matched patient encounter examples with publicly available healthcare payor cost data to determine an estimated health plan cost savings per patient [data not shown]. These patient care stories and projected cost savings were reported back to the payor and PPCN pharmacists as a part of a rapid reporting mechanism and value proposition. Ultimately, the value of the patient care services provided led to a second contract opportunity with the Medicaid MCO for continued payment for CMM by PPCN pharmacists.

## 3. Implementation

### 3.1. Applying an Implementation Framework to the QET Project

The Active Implementation Frameworks (AIFs) are an evidence-based collection of frameworks designed to support the successful implementation of an innovation. Historically, these frameworks were used in education implementation practice and research, but more recently have been applied to healthcare innovations [15]. The five core components of the AIFs are (1) usable innovations, (2) implementation stages, (3) implementation drivers, (4) implementation cycles, and (5) implementation teams [16]. The AIFs are used to guide implementation processes, and when applied to our efforts with the QET project, they provide a helpful framework for others to replicate and adapt this student engagement strategy to support a local CPESN network.

The implementation stages component of the AIFs is further categorized into 4 stages: (1) exploration; (2) installation; (3) initial implementation; and (4) full implementation [16]. The work of the QET specifically focused on support of initial implementation. Although each of these implementation stages can happen simultaneously during the implementation of an innovation, they provide a relatively linear description of the implementation of this payor contract for CMM [15,16]. For example, PPCN and University of Pittsburgh leadership prepared for implementation in the exploration and installation stages by identifying a “usable innovation” (i.e., CMM), then forming “implementation teams,” and finally recruiting and training pharmacists to provide CMM. The QET was one implementation team that was formed just before the initial implementation stage.

The QET supported the initial implementation by utilizing the core AIF component of “implementation cycles.” Specifically, the students engaged in rapid-cycle problem solving by facilitating information gathering and problem solving during the early days of CMM implementation at each of the pharmacies. Rapid-cycle problem-solving is an improvement cycle within the “Plan, Do, Study, Act” quality improvement process, and is used to quickly solve problems that emerge during the rollout of an innovation [17]. Structuring the work of the QET around this improvement cycle was helpful to the QET students and the leadership team guiding them. This cyclical structure focused student efforts and facilitated the provision of ongoing support and feedback from PPCN and CLIP leadership to the students throughout their work. The QET students simultaneously learned about improvement cycle theory while performing it during this “real world” experience.

Since the initial payor contract between PPCN, STRAND and the Pennsylvania Medicaid MCO in 2017, the program contract has been renewed annually, with the most recent contract period beginning in February 2020. Efforts to reach full implementation are ongoing as new PPCN pharmacies join the network and sign onto the contract each year. The QET is an agile implementation team that continues to support pharmacies presently. The QET activities and structure have adapted over time in response to implementation needs throughout subsequent contract periods in the ongoing efforts towards sustainment.

### 3.2. Implementation Strategies Utilized by the QET

The implementation strategies most frequently utilized by the QET can be defined using four standardized implementation strategy definitions, outlined in the Expert Recommendations for Implementing Change (ERIC) project [18]. These include: (1) facilitation; (2) capture and share local knowledge; (3) remind clinicians; and (4) purposely reexamine the implementation. The student outreach calls to pharmacies represented the facilitation implementation strategy. This strategy involves interactive problem solving between parties of a supportive interpersonal relationship to address an area for improvement [18]. The earlier examples of rapid-cycle problem solving concerning the addition of health conditions into the documentation platform and the creation of a pediatric patient guide (see Section 2.2) highlight how the QET utilized facilitation. The QET also captured and shared local knowledge between the pharmacies. Members of the QET collected ideas during phone calls and directly communicated this information between the pharmacies and with PPCN leadership. PPCN leadership would then disseminate the most useful information to the entire network. Sharing local knowledge and learning from other pharmacies’ experiences assisted PPCN pharmacists with developing and adapting implementation strategies for CMM within the contexts of their own pharmacies. The QET also served to remind clinicians about CMM goals during their biweekly phone calls. For example, if a pharmacist had not yet initiated the provision of CMM through the contract, or was slow to implement CMM, then the QET member’s phone call could serve as a reminder to begin or continue the implementation process. Lastly, the QET aided in purposely reexamining the implementation throughout the initial implementation process by monitoring the needs, progress and preferences of the pharmacists while initial implementation was ongoing. For example, one component of the implementation effort was direct communication between the Executive Manager of PPCN and the participating pharmacies. Pharmacists provided feedback to the QET that this open line of communication was helpful to them, which supported the continuation of this practice during and beyond initial implementation. We recommend utilizing these four implementation strategies to those who wish to involve students in the implementation of medication management services through a CPESN network.

## 4. Outcomes

### 4.1. QET Project Outcomes

The QET students completed a total of 155 interviews with 48 pharmacists. These pharmacists represented 59 of the 61 independent pharmacies that signed the payor contract for CMM. Some of the pharmacies in the cohort had a shared pharmacist contact (i.e., one pharmacist who worked for multiple pharmacies), resulting in a fewer number of pharmacists interviewed than the number of pharmacies included in the initial interview cohort. Additionally, though the QET students made biweekly call attempts to each of the pharmacy contacts involved, not every call resulted in a successful semi-structured interview. The interviews ranged in duration from approximately 4 to 21.5 min and averaged approximately 9.5 min per call. A qualitative analysis of interview transcripts is planned.

### 4.2. Adoption of CMM at Participating Pharmacies

**Figure 1:**
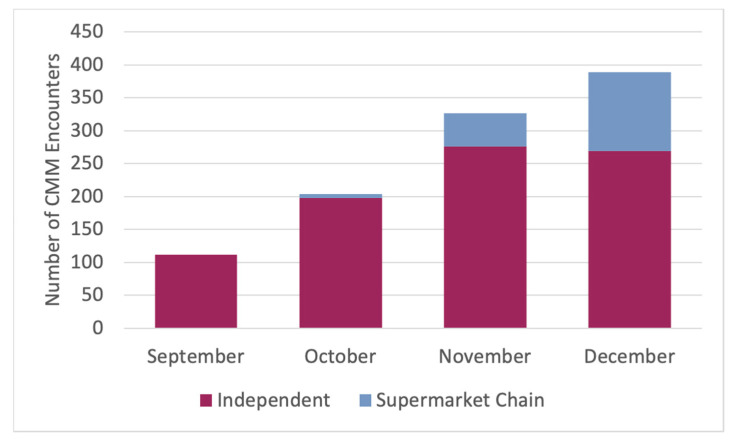
Pennsylvania Pharmacists Care Network Comprehensive Medication Management Patient Encounter Data for 4-Month Pilot Program, 2017.

In total, 1031 CMM patient encounters were completed by PPCN pharmacists during the initial four-month contract period. For this variable, the participating pharmacies included both independent and supermarket chain pharmacies. Figure 1 depicts the increase in encounters over the first 4 months, demonstrating a positive progression in program adoption. Of these 1031 patient CMM encounters, 855 were completed at the independent pharmacies, and 176 were completed at the 16 regional supermarket chain pharmacies that also participated in the contract. Of note, the supermarket chain pharmacy contract period began in October 2017, and a support process similar to that of the QET was implemented in these pharmacies after that time. Details regarding the implementation of this payor contract for CMM at the supermarket chain pharmacies are reported separately [19]. The total of 1031 CMM encounters represents, on average, 258 CMM encounters per month for participating pharmacies.

## 5. Discussion

There is a growing body of literature on CPESN networks and the implementation of medication management services within community pharmacies [20,21,22,23,24,25,26,27]. Though there is a growing body of literature on these topics, to our knowledge, formally utilizing students as an implementation team for a CPESN network is a novel approach. In this paper, we described the establishment of a student pharmacist-led implementation team, the QET, which assisted with the initial implementation of a payor contract for CMM in independent community pharmacies as part of a statewide CIN. The activities of the QET included biweekly phone calls and communications with the PPCN, CLIP Center and STRAND leadership throughout the contract period. These efforts contributed to the adoption of CMM during the contract period, as demonstrated by the increase in the number of patient encounters completed by participating pharmacies each month. The efforts of the QET students helped ensure the quality of the program and created a mechanism to engage pharmacists who participated in the program. Because this experience was built into the students’ ARCO within the PharmD curriculum, they were motivated to complete the work. Integration into the curriculum also provided an infrastructure for staff and faculty support of the project.

The relationships and conversations between the QET students and pharmacists fostered powerful collaborative learning surrounding the implementation of the payor contract for CMM. Because the same students called the same pharmacies throughout the contract period, mentoring relationships readily formed. Pharmacists provided information to the students on best practices as well as the barriers they faced when implementing the network contract services. PPCN and CLIP Center leadership gathered anecdotal evidence that pharmacists felt they contributed to the students’ development as pharmacists during this time. The patient stories the pharmacists shared with the students also became insightful patient experiences to share back to the payor. The stories captured both the quality of care provided and permitted cost savings estimates [data not shown]. Both of these outcomes were important to the payor and supported justification for the renewal of the contract, which is now in its fourth year. Utilizing a team of student pharmacists as a component of an implementation approach proved incredibly valuable to the network. It also provided a meaningful learning experience for the students who participated [28]. This structure kept PPCN pharmacists engaged, demonstrated desirable outcomes to the payor, and ultimately supported the provision of CMM by PPCN pharmacists to patients.

One limitation is that reporting on this project after initial implementation limited our ability to collect data points that might be valuable for the replication of the project by others. For example, we did not systematically collect time spent by students and faculty mentors on the project, which limited our ability to report on resources that others would need to replicate the program. Additionally, the University of Pittsburgh School of Pharmacy had a well-established relationship with the CPESN network, PPCN, which permitted the ease of communication between parties. This may limit generalizability to other institutions and CPESN networks that may not have the same structure or existing relationships.

Since its inception, the QET has sustained and adapted to meet the needs of PPCN. An increasing number of students support a growing number of PPCN pharmacies that participate in the Medicaid MCO contract for CMM. The QET now includes student pharmacists from the second, third and fourth professional years of the PharmD program. As of 2018, the team is led by a CLIP Center Fellow, which provides a community of learning experience and additional educational and leadership benefits to post-graduate trainees [29]. The QET students now support all PPCN pharmacies regardless of payor contract status through phone call check-ins and emails with reminders and resources related to community practice transformation. There are now over 140 pharmacies in PPCN, 80 of which are participating in the Medicaid MCO contract in 2020. The QET continues to collect patient stories from PPCN pharmacists to help PPCN leadership advocate for new programs with additional payors. Notably, nearly all of the work of the QET to-date has been done remotely. Remote support that produces meaningful results and learning for students is now more relevant than ever in light of the COVID-19 pandemic [30].

Utilizing student learners to support community pharmacies opens the door for new student engagement opportunities in community pharmacy practice transformation. Involving student pharmacists in practice transformation has become a national topic of conversation amidst the recent national efforts to unite colleges/schools of pharmacy with CPESN networks for practice transformation initiatives. For example, the Academia-CPESN Transformation (ACT) Pharmacy Collaborative was formed in 2019 to unite the efforts and resources of CINs of community pharmacies and colleges/schools of pharmacy [31]. The ACT Pharmacy Collaborative includes over 250 faculty nationwide at 88 colleges/schools of pharmacy and provides a platform to further explore and discuss how student pharmacists can support community pharmacy practice transformation. The QET project is one example of how an individual school of pharmacy and its students worked collaboratively with a local CPESN network.

## 6. Conclusions

Student pharmacists can be a powerful asset in the implementation of payor programs within CINs of community pharmacies. The use of the AIFs and select key implementation strategies can effectively guide and design student involvement in quality improvement work. Student engagement in quality improvement, when supported with faculty and staff mentorship, can be instrumental in payor contract implementation, while simultaneously supporting student learning.

## Figures and Tables

**Table 1:** Sample Interview Questions for Student-Led Pharmacist Interviews.

Category	Sample Questions
Comprehensive Medication Management (CMM) Workflow	How do the CMM activities fit into your daily workflow? What types of patient encounters (e.g., initial vs. follow-up; scheduled vs. unscheduled) are you conducting most frequently?
Team Involvement in CMM	What members of your team are inviting patients to participate? What members of your team are involved in documenting patient outreach and patient care activities? How are other non-pharmacist team members involved in the patient care process?
Patient Engagement	How are you identifying and inviting patients for the patient care services? Describe a specific example to illustrate how your patients are receiving the program. What engagement strategies are you utilizing to involve patients?
Successful Strategies and Implementation Support	What strategies do you believe lead to success with the program? What additional support do you need to have greater success with this program? What advice can you offer us and other pharmacies for implementation?
